# Apoptosis-like cell death pathways in the unicellular parasite *Toxoplasma gondii* following treatment with apoptosis inducers and chemotherapeutic agents: a proof-of-concept study

**DOI:** 10.1007/s10495-013-0832-8

**Published:** 2013-03-07

**Authors:** Ayu Dewi Ni Nyoman, Carsten G. K. Lüder

**Affiliations:** 1 Institute for Medical Microbiology, University Medical Center, Georg-August-University, Kreuzbergring 57, 37075, Göttingen, Germany; 2 Department of Biochemistry, Faculty of Medicine and Health Sciences, Udayana University, Sudirman Denpasar, 80232 Bali, Indonesia

**Keywords:** *Toxoplasma gondii*, Unicellular parasite, Apoptosis, Therapeutics, Mitochondria, Protease

## Abstract

Ancient pathways of an apoptosis-like cell death have been identified in unicellular eukaryotes including protozoan parasites. Here, we examined programmed cell death in the apicomplexan *Toxoplasma gondii* which is a common intracellular pathogen of humans and warm-blooded animals. Treatment of extracellular *T. gondii* with various pro-apoptotic stimuli significantly induced DNA strand breaks as revealed by TUNEL and flow cytometry. Using staurosporine or miltefosine as pro-apoptotic stimuli, parasites also presented a reduced cell size, i.e. pyknosis and externalized phosphatidylserine while the plasma membrane remained intact. Importantly, staurosporine also induced DNA strand breaks in intracellular *T. gondii*. Data mining of the *Toxoplasma* genome resource identified 17 putative cell death-associated genes encoding proteases, a nuclease and several apoptosis regulators. Staurosporine-treated parasites but not controls strongly up-regulated several of these genes in a time-dependent fashion with a putative PDCD2 protein being more than 100-fold up-regulated. However, the mitochondrial membrane potential (Δ*Ψ*_m_) remained intact and caspase-like activity increased only slightly during staurosporine-triggered cell death. As compared to staurosporine, the transcriptional response of parasites to miltefosine was more restricted but PDCD2 was again strongly induced. Furthermore, *T. gondii* lost their Δ*Ψ*_m_ and rapidly presented strong caspase-like activity during miltefosine treatment. Consequently, protease inhibitors abrogated miltefosine-induced but not staurosporine-induced *Toxoplasma* cell death. Finally, toxoplasmacidal drugs triggered DNA strand breaks in extracellular *T. gondii*. Interestingly, clindamycin also induced markers of an apoptosis-like cell death in intracellular parasites. Together, the data indicate that *T. gondii* possesses ancient apoptosis-like cell death machinery which can be triggered by chemotherapeutic agents.

## Introduction

Apoptosis is a form of programmed cell death (PCD) that until recently was thought to be confined to metazoans. A form of cellular suicide in single-celled organisms indeed appeared counter-intuitive and evolutionary not favorable. This view has been challenged with the detection of markers that are characteristic for apoptosis in metazoans in several unicellular organisms including protozoan parasites [1]. Signs of apoptosis have been recognized in parasites from such different phylogenetic branches as the kinetoplastids, the apicomplexans, the amoebozoa, *Blastocystis*, the trichomonads and the diplomonads suggesting an ancient origin of death-regulating genes during the evolution of eukaryotes [2–4]. Hallmarks of apoptosis in these organisms include shrinkage and rounding-up of the cell, externalization of phosphatidylserine (PS) from the inner to the outer leaflet of the cell membrane while maintaining membrane integrity, and DNA fragmentation as well as chromatin condensation [1]. In silico analysis of genome databases of various protists [5] and evolutionary considerations [2, 3, 6] also argue for the existence of ancient PCD pathways in unicellular organisms. Although apoptosis-like cell death in protozoa shares several characteristic features of apoptosis in metazoans, the underlying mechanisms clearly differ. For example, cysteine peptidases with specificity for aspartate, i.e. caspases, are key players of apoptosis in higher eukaryotes but are not present in the genomes of protists [5]. Likewise, members of the Bcl-2 family which sense and integrate pro-apoptotic signals into the mitochondrial apoptotic pathway in mammals are absent from protozoa. Thus, the pathways that regulate the demise of protozoa differ from those present in mammals. This makes apoptosis in protozoa a promising target for the development of novel intervention strategies against parasites of medical and veterinary importance.

*Toxoplasma gondii* is an obligatory intracellular parasite of the phylum Apicomplexa. It is ubiquitously distributed and infects warm-blooded wild and domestic animals as well as up to 30 % of the worlds’ human population [7]. Following infection, rapidly replicating tachyzoites infect a variety of different cell types and disseminate throughout their host leading to the acute phase of infection. Although infection of immunocompetent hosts is usually benign to asymptomatic, the parasite is not eradicated by the hosts’ immune defense. It instead transforms into the dormant bradyzoite stage which is able to persist for the hosts’ life span within intracellular cysts mostly within neural and muscular tissues [8]. In immunocompromised hosts, i.e. AIDS patients or transplant recipients, primary infection or—more commonly—reactivation of persistent infection can lead to life-threatening toxoplasmosis due to uncontrolled parasite replication and tissue damage [9]. Likewise, after vertical transmission of the parasite during acute infection of pregnant women to their offspring, uncontrolled parasite replication can lead to congenital toxoplasmosis ranging from stillbirth to severe symptoms at birth or sequelae including retinochorioiditis or mental retardation. Ocular toxoplasmosis can also result from postnatal infection, and *T. gondii* has been recognized as the most common pathogen leading to posterior uveitis in immunocompetent patients [10].

Chemotherapy of symptomatic toxoplasmosis is mandatory; however, available drug regimens including pyrimethamine plus sulfadiazine, clindamycin, and atovaquone are limited and are only effective against the replicating tachyzoite stage [9]. Furthermore, side effects of the available drug treatments are common, and treatment failures have been reported which may result from strain-specific differences in susceptibility [11]. Thus, the development of novel drugs against toxoplasmosis is critical, as is a better understanding of molecular pathways which regulate cell death in *T. gondii*.

In previous studies we have extensively characterized the ability of intracellular *T. gondii* parasites to inhibit apoptosis of its host cell [12–14]. In these studies we recognized the occurrence of extracellular parasites which were positive for DNA strand breaks as determined by terminal deoxynucleotidyl transferase-mediated dUTP nick end labelling (TUNEL) (Lüder, unpublished observation), i.e. a widely used marker for apoptotic cells [15]. This prompted us to investigate whether and to what extent apoptosis-like cell death occurs in *T. gondii* under different environmental conditions and how this might be regulated. Our results show that extracellular and intracellular parasites undergo a form of programmed cell death that share several common features with metazoan apoptosis. In silico analysis and biochemical data revealed components of apoptosis-like cell death pathways in *T. gondii* that were triggered by common pro-apoptotic stimuli. Finally we present evidence that apoptosis-like cell death can be triggered in intracellular parasites under clindamycin treatment. Together, the results raise the intriguing possibility that apoptosis-like cell death pathways can be employed in order to combat toxoplasmosis.

## Materials and methods

### Parasites, in vitro cultivation and induction of cell death

The mouse-avirulent type II *T. gondii* strain NTE [16] was used for all experiments. Parasites were propagated in L929 murine fibroblasts as host cells; co-cultures were cultivated in Roswell Park Memorial Institute (RPMI) 1640 medium supplemented with 1 % heat-inactivated fetal bovine serum (FCS), 100 U/ml penicillin and 100 μg/ml streptomycin. For infection assays, human foreskin fibroblasts (HFF) were used as host cells, and they were cultivated in Dulbecco’s Modified Eagle Medium (DMEM) supplemented with 10 % FCS, 1 mM sodium pyruvate, non-essential amino acids and antibiotics as above. Cells were grown at 37 °C in a humidified 5 % CO_2_ atmosphere. For induction of cell death in extracellular parasites or for infection assays, parasites were isolated from L929 co-cultures by differential centrifugation and thoroughly washed [12]. Briefly, host cells were pelleted by centrifugation at 35×*g* for 5 min. The supernatant was then centrifuged at 1,350×*g* for 10 min, the parasites were washed twice, resuspended in culture medium and counted in a hemocytometer grid. For induction of cell death, 2 × 10^7^ parasites were seeded in 0.5 ml of culture medium and were treated for up to 72 h with 5–10 μM staurosporine, 20 μM miltefosine, 2 mM H_2_O_2_, or 5 μM camptothecin (all chemicals from Sigma-Aldrich, Taufkirchen, Germany). Alternatively, 10^5^ parasites per well were used to infect confluent HFF cells that were grown in 24-well plates containing cover slips. Twenty-four hours after infection, infected HFF cells were treated with 10 μM staurosporine or 20 μM miltefosine for 3–48 h. In some experiments, 100 μM Z-VAD-FMK (Enzo Life Sciences, Lörrach, Germany) or 100 μM E64 (Sigma-Aldrich) were added 1 h prior to the treatment with pro-apoptotic stimuli.

### Treatment of *T. gondii* with parasiticidal drugs

HFF monolayers were infected as described above. After removal of extracellular parasites at 4 h post infection, infected cells were incubated for 24 and 48 h in medium containing 100 nM atovaquone, 500 nM clindamycin, 1 μM pyrimethamine or 500 nM anisomycin (all from Sigma-Aldrich). Since clindamycin exerts toxoplasmacidal activity during the second round of intracellular replication [17], parasites that emerged from the drug-treated cells within 3–4 days p.i. were centrifuged at 1,500×*g* for 10 min, and 25 % of the parasites were used to infect fresh HFF cells in the continuous presence of clindamycin (2nd cycle of treatment).

### TUNEL assay and flow cytometry

DNA strand breaks were detected in freshly isolated *T. gondii* parasites and those treated with pro-apoptotic stimuli or the vehicle alone (mock treatment) using a terminal deoxynucleotidyl transferase-mediated dUTP nick end labelling (TUNEL) assay as recommended by the manufacturer (Roche, Mannheim, Germany). After harvesting the parasites by centrifugation at 1,800×*g* for 10 min, they were washed in PBS, pH 7.4, and were then fixed in 4 % (w/v) paraformaldehyde in 0.1 M sodium cacodylate, pH 7.4 for 1 h at room temperature. After having been washed, the cells were permeabilized for 5 min with 0.1 % Triton X-100 in 0.1 % sodium citrate. They were then centrifuged (as above) and were incubated in TUNEL reaction mix containing fluorescein-conjugated dUTP and terminal deoxynucleotidyl transferase for 1 h at 37 °C in the dark. Thereafter, cells were washed in PBS and were analysed in a FACSCalibur flow cytometer (BD Biosciences, San Diego, CA, USA). A total of 50,000 cells were recorded from each sample.

### Phosphatidylserine exposure

Translocation of phosphatidylserine from the inner to the outer leaflet of the plasma membrane of *T. gondii* was detected using the Annexin V-PE apoptosis detection kit (BD Biosciences, Heidelberg, Germany). Parasites were harvested by centrifugation (see above), were washed twice in ice-cold PBS, and 1 × 10^6^ parasites/ml were then resuspended in 1× Annexin V binding buffer. After addition of 5 μl of Annexin V solution and/or 5 μl of 7-AAD solution per 100 μl, cells were incubated for 15 min at room temperature in the dark. Thereafter, 400 μl of 1× Annexin V binding buffer were added, and 10,000 cells of each sample were analysed by FACS (FACSCalibur). Unstained cells, cells stained with Annexin V alone, and cells stained with 7-AAD alone were used as controls to adjust channel compensation.

### Determination of mitochondrial membrane potential (Δ*Ψ*_m_)

In order to determine the Δ*Ψ*_m_, extracellular parasites were harvested by centrifugation, were washed and were then incubated in 500 nM MitoTracker^®^ probe Orange CM-H_2_TMRos (Life Technologies/Molecular Probes, Darmstadt, Germany) in RPMI 1640 for 45 min at 37 °C. After having been washed in PBS, cells were fixed in 4 % paraformaldehyde, 0.1 M sodium cacodylate, pH 7.4 for 15 min. Parasites were embedded in Mowiol 4-88 mounting medium (Calbiochem, Schwalbach, Germany) and at least 500 cells of each sample were examined by using a Leica TCS SP2 confocal laser scanning microscope.

### Detection of caspase-like activities

CaspaTag™ pan-caspase in situ assay kit (Chemicon/Millipore, Schwalbach, Germany) was used to detect the activity of caspase-like proteases during cell death induction in *T. gondii*. To this end, extracellular parasites were incubated in the presence of 10 μM staurosporine or 20 μM miltefosine for 2, 4, 24 and 48 h to induce cell death or were left untreated. At indicated time points, treated parasites or freshly isolated ones were mixed with fluorescein-conjugated FAM-VAD-FMK (FLICA reagent) for 1 h at 37 °C. After having been washed three times with washing buffer, parasites were analysed by flow cytometry (FACSCalibur).

### Immunofluorescence staining, TUNEL assay and fluorescence microscopy

DNA fragmentation in intracellular *T. gondii* or in isolated parasites was morphologically visualized by double fluorescence microscopy. *Toxoplasma*-infected HFF cells grown on glass cover slips, or extracellular parasites which had been spread onto glass slides and air-dried, were fixed in 4 % (w/v) paraformaldehyde in 0.1 M sodium cacodylate, pH 7.4 for 1 h. Nonspecific binding sites were blocked, and cells were permeabilized for 1 h in 0.1 mg/ml saponin, 1 % bovine serum albumin (Sigma-Aldrich, Taufkirchen, Germany) in PBS (blocking buffer). After having been washed in 0.1 mg/ml saponin in PBS, the cells were incubated in rabbit anti-*Toxoplasma* hyperimmune serum diluted in blocking buffer. After washing, immune complexes were labelled for 1 h with 0.95 μg/ml Cy3-conjugated donkey anti-rabbit IgG (Dianova, Hamburg, Germany) in blocking buffer. Cells were washed again, were treated with 0.1 % Triton X-100 in 0.1 % sodium citrate for 5 min, and were then covered with TUNEL reaction mix (in situ cell death detection kit, fluorescein; Roche, Mannheim, Germany) for 1 h at 37 °C in the dark. In some experiments, parasite nuclei were additionally stained for 45 min with 2 μg/ml Hoechst 33258 diluted in saponin/PBS. After having been washed in PBS, cells were mounted using Mowiol 4-88 and examined by confocal laser scanning microscopy (Leica TCS SP2). For determining the extent of DNA fragmentation, at least 500 parasites per sample were inspected. Parasite replication was determined by counting the number of tachyzoites of at least 100 parasitophorous vacuoles.

### RNA isolation and quantitative RT-PCR

Total RNA was extracted from 7 × 10^7^ parasites treated or not with pro-apoptotic stimuli using the GenElute™ mammalian total RNA kit as recommended by the manufacturer (Sigma-Aldrich). Contaminating DNA was digested by using DNase 1 (amplification grade; Sigma-Aldrich). Up to 2 ug of mRNA were then reverse transcribed for 90 min at 37 °C by oligodT priming using the Omniscript^®^ reverse transcription kit (Qiagen, Hilden, Germany). Serial dilutions of cDNA encoding for putative cell death regulators and executors were amplified by quantitative real time PCR in a capillary-based LightCycler^®^ system (Roche, Mannheim, Germany) using the FastStart DNA Master^PLUS^ SYBR Green I kit (Roche) and 0.25 μM each of the primers specified in Table 1. For 20 μl PCR reaction, the following components were mixed: 2 μl of cDNA, 0.25 μM of forward and reverse primers, 4 μl of buffer and enzyme mixture (LightCycler^®^ FastStart DNA Master^PLUS^ SYBR Green I, Roche Applied Science, Germany). The relative gene expression levels were calculated as the fold change between freshly isolated (control) and treated (staurosporine, miltefosine, or mock-treatment) *T. gondii* using the formula Normalized ratio_(treated/freshly)_ = (*E*_target_)^ΔCP(freshly-treated)^/(*E*_actin_)^ΔCP(freshly-treated)^, where amplification of actin was used to normalize between the different samples and target were putative cell death-associated genes.

**Table 1:** Primers for quantitative real time PCR of *T. gondii* putative cell death-related cDNAs

Gene ID	Forward (5′–3′)	Reverse (5′–3′)	Amplicon (bp)
TgME49_008710	AGGCAGCGAAACACAGAAAG	ACATGCAGGGAAGCTCCA	264
TgME49_007620	CGCGTACCCATACGTGAAGA	GCGACGAACTGTTGCTTGTC	234
TgME49_078970	ACTTCGTCGCGTTTCACTTT	ATGCAGTACGCATTGAGCAG	248
TgME49_053000	AAGGCGAGAGGAATGGATTT	CGTCGTAAAATTGCAGCAGA	201
TgME49_094420	AGAGAGGAGGGAGACGAAGG	CAGCGAACTGTGTTCGTCAT	156
TgME49_093820	GACGATTCACACATGGTTGC	TTGATTTTCCTGGGAGAACG	228
TgME49_093830	GACCTCTCGGCTGTAGCATC	CGCACCGTCTGTCAGAGTTA	158
TgME49_021360	CTTCGTCAGCGGGAATATGT	GCCGATAGTGAGTTGGGTGT	241
TgME49_049770	GCCATTGTTCCACGTCTTTT	GAAGGGGAAGGAAGGAGATG	162
TgME49_109560	CCAGGACAATTTGCACACAC	CGTCCGCTCTTTTCTTTACG	232
TgME49_003030	CCAGGCTGCTACTCCTTACG	GAACCCGTTTCAATGTCGAT	171
TgME49_105490	TCTTGCCAGCGTGAATACTG	GCGCTGATAGTCACTCCACA	247
TgME49_105870	GATGCATTCTGTGCGTGTCT	GGGACTCACCTGGATTCTGA	159
TgME49_035560	TCCCAGTCCACAGAGTTCCT	AGAGACGGCAAAGGATCAGA	209
TgME49_107780	GTTGCTGGGATAGCTTCTGC	GCCTTGAGAGGACAGAGGTG	240
TgME49_035880	CCGAGCGAAGTTTTTACGTC	CACCGAAAATGTGAGACACG	170
TgME49_006490	TTCGTCCACTACGCTTGTTG	GGGAGAATCGTTTCGTCGTA	207
TgME49_009030 (actin)	TGGCAACGAGCGATTCCGCTG	GTTCCTTGGTCAGCCTCTCGCC	200

### Statistical analysis

Results are expressed as mean ± SEM of at least three independent experiments unless otherwise stated. Significant differences between experimental groups were identified by Student’s *t* test. *P* values of less than 0.05 were considered significant.

## Results

### In situ DNA fragmentation in *T. gondii*

**Fig. 1:**
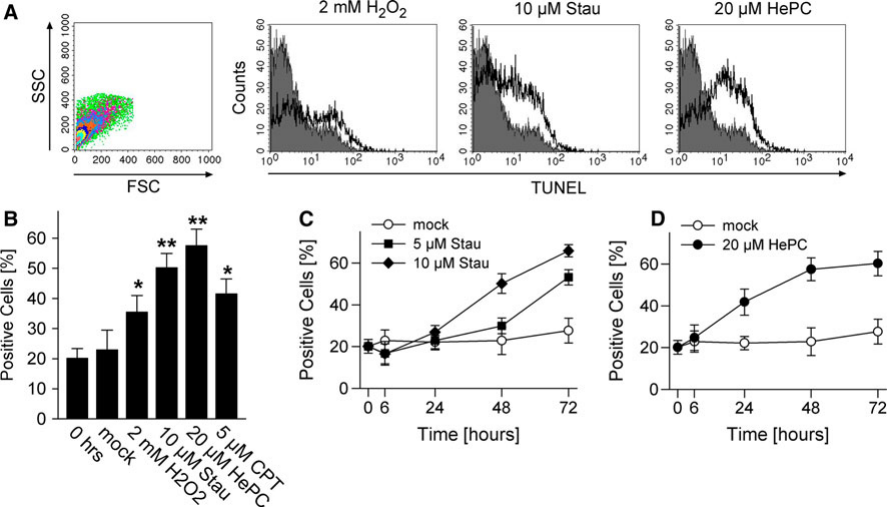
Pro-apoptotic stimuli trigger DNA fragmentation in *T. gondii*. Parasites were treated with hydrogen peroxide (H_2_O_2_), staurosporine (Stau), miltefosine (HePC) or camptothecin (CPT) as indicated, or were mock-treated. At different time points after treatment, DNA strand breaks were visualized by in situ TUNEL labelling and parasites were analysed by flow cytometry. Freshly isolated parasites were processed in parallel (0 h). **a** Parasites were selected according to FSC/SSC parameters and were analysed for terminal deoxynucleotidyl transferase-mediated dUTP nick end labelling (TUNEL). The *graphs* show representative histograms from parasites which have been treated with the indicated stimuli for 48 h (*open histograms*), or those which have been freshly isolated (*filled histograms*). **b** Mean percentages ± SEM of TUNEL-positive *T. gondii* as determined by flow cytometry; results are from 5 to 6 independent experiments (CPT: *n* = 2). Significant differences between treated *T. gondii* and untreated controls are indicated by one (*p* < 0.05) or two *asterisks* (*p* < 0.01). **c, d** Kinetics of DNA fragmentation in *T. gondii* following treatment with staurosporine or miltefosine or following mock treatment. Data represents mean ± SEM from at least three independent experiments

DNA fragmentation is one of the common biochemical downstream hallmarks of apoptosis in mammals [18] and is considered an important marker for apoptosis-like cell death in protozoa [1]. In situ TUNEL labelling and flow cytometry revealed that a population of freshly isolated *T. gondii* contained ~20 % of TUNEL-positive parasites and this did not significantly change within the next 48 h (Fig. 1). Treatment of *T. gondii* with H_2_O_2_, which triggers apoptosis in mammalian cells, yeast and protozoa [19–22], augmented the percentage of TUNEL-positive parasites moderately (Fig. 1a, b) (*p* < 0.05, Student’s *t* test), indicating that oxidative stress may promote cell death in *T. gondii*. Remarkably, the protein kinase inhibitor staurosporine and the leishmanicidal alkylphosphocholine drug miltefosine (HePC) strongly induced DNA fragmentation in 50 and 57.5 % of parasites, respectively (Fig. 1a, b) (*p* < 0.0005). The ATP analog staurosporine is a highly potent and widely used stimulus of cell-intrinsic apoptotic pathways [23]. Likewise, miltefosine exerts pro-apoptotic activity against cancer cells and *Leishmania* spp., possibly via perturbation of fatty acid and sterol metabolism, mitochondrial dysfunction and/or inhibition of the Akt/PKB pathway [24]. Camptothecin (CPT), a DNA topoisomerase I inhibitor, also triggered DNA fragmentation in *T. gondii* (Fig. 1b) (*p* < 0.05). In *Leishmania* spp., CPT activates a cell death pathway via deregulation of the mitochondrium [25], thus implying that also in *T. gondii*, impaired mitochondrial function might trigger a cell death pathway which finally leads to DNA fragmentation.

Due to their well-established pro-apoptotic activities and the strong induction of DNA fragmentation in *T. gondii* (see above), we concentrated on staurosporine and miltefosine in the following experiments. Kinetics of DNA fragmentation revealed a time-dependent increase in the percentage of TUNEL-positive parasites from 24 h onwards until 72 h post staurosporine treatment (Fig. 1c). Furthermore, staurosporine triggered DNA strand breaks in a dose-dependent manner (Fig. 1c). Following treatment with miltefosine, a significant increase in DNA fragmentation was already observed at 24 h post treatment and reached a plateau at 48 h (Fig. 1d).

**Fig. 2:**
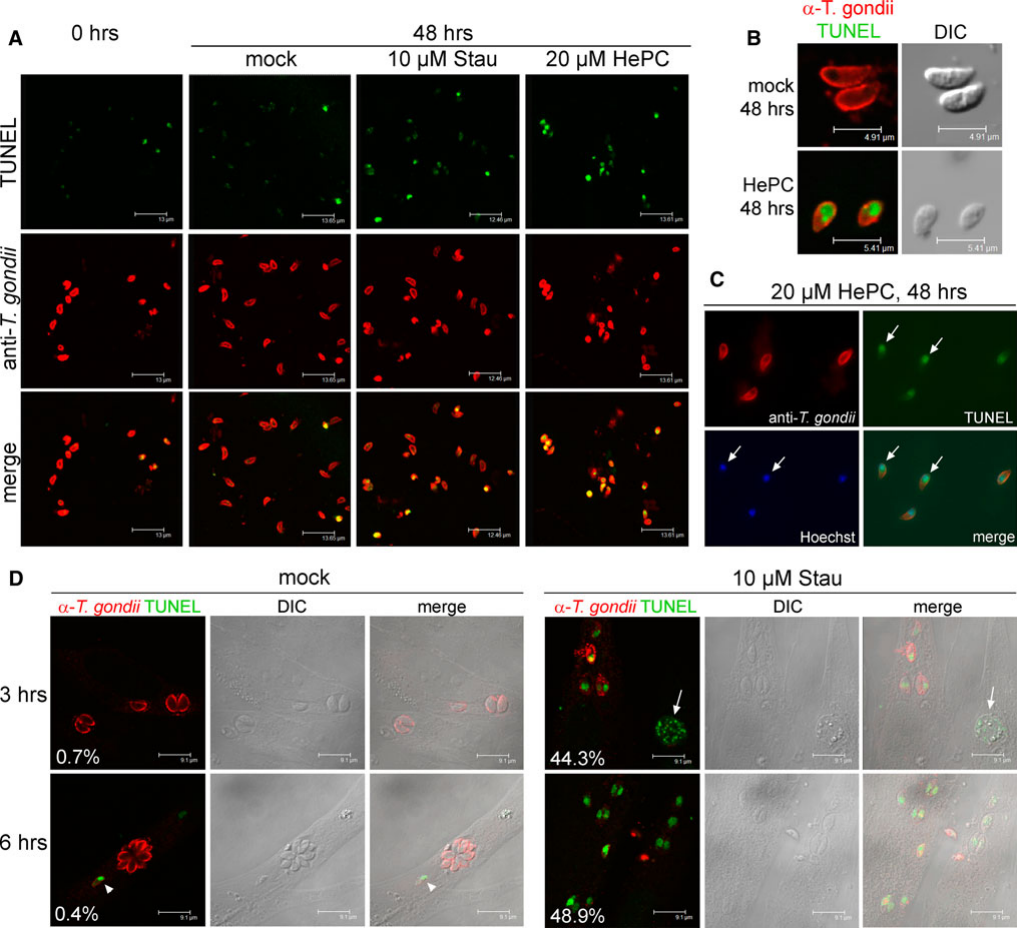
DNA strand breaks and morphology of *T. gondii* following treatment of extracellular (**a–c**) or intracellular (**d**) parasites with pro-apoptotic stimuli and in situ TUNEL labelling. Parasites were treated with staurosporine (Stau), miltefosine (HePC), or were mock-treated for 3, 6 or 48 h as indicated, or were left untreated (0 h). After labelling with anti-*Toxoplasma* serum (*red fluorescence*) and DNA strand breaks using a terminal deoxynucleotidyl transferase-mediated dUTP nick end labelling (TUNEL; *green fluorescence*), cells were examined by confocal laser scanning microscopy. Differential interference contrast (DIC) images were recorded to verify the morphology of extracellular parasites (**b**) or to confirm the localization of intracellular parasites (**d**). *Arrowhead* and *arrow* in **d** indicate a TUNEL-positive intracellular parasite after mock-treatment and a TUNEL-positive host cells after Stau treatment, respectively. Data indicate the percentage of parasitophorous vacuoles containing TUNEL-positive parasites. Partially, parasite nuclei were visualized in parallel using the nuclear dye Hoechst 33258 (*blue fluorescence*) and conventional fluorescence microscopy (**c**). *Arrows* in **c** indicate co-localization of TUNEL-positive organelles with parasite nuclei. Representative micrographs from three independent experiments are depicted

Confocal laser scanning microscopy of extracellular *T. gondii* which had been labelled using parasite-specific antibodies and a TUNEL reaction mix confirmed the increase in TUNEL-positive parasites following treatment with staurosporine and miltefosine as compared to freshly isolated controls and mock treated controls (Fig. 2a). Both the size and position of the TUNEL-reactive structures were compatible with staining of the parasite nuclei (Fig. 2a, b). By using the nuclear dye Hoechst 33258, we confirmed that TUNEL-positive organelles in parasites which had been treated with a pro-apoptotic stimulus co-localized with the parasite nuclei (Fig. 2c; arrows). TUNEL-positive parasites were mostly round-shaped and smaller than the regular TUNEL-negative crescent-shaped parasites (Fig. 2a, b). Both rounding-up of the cell and reduction of the cell volume are characteristic features of cells undergoing apoptosis [18].

Due to the parasites’ intracellular life style, we then tested whether *T. gondii* within its natural environment also shows signs of apoptosis upon treatment with pro-apoptotic stimuli. To this end, parasite-infected HFF cells were treated with staurosporine or miltefosine for various time periods. Remarkably, treatment with staurosporine for 3–6 h induced significant levels of DNA strand breaks in intracellular parasites as determined by TUNEL assay (44.3 and 48.9 % at 3 and 6 h after treatment, respectively; Fig. 2d). Due to the induction of apoptosis in the host cells (Fig. 2d, arrow), we were not able to follow up induction of DNA strand breaks in *T. gondii* beyond 6 h of treatment. TUNEL-positive intracellular parasites were not regularly detected in mock-treated cells; however, it is interesting to note that occasionally, we also observed DNA strand breaks in intracellular control parasites (Fig. 2d, arrowhead). Miltefosine did not induce DNA strand breaks in intracellular parasites (0.6 and 0.5 % at 3 and 6 h after treatment, respectively). Collectively, these data established that treatment of both extracellular and intracellular *T. gondii* with well-known pro-apoptotic stimuli induces significant levels of DNA fragmentation, i.e. a hallmark of apoptosis. We also provide compelling evidence that extracellular *T. gondii* parasites are highly vulnerable to miltefosine, a drug that at least partly exerts its activity by triggering apoptosis.

### *Toxoplasma gondii* displays multiple markers of apoptosis after treatment with pro-apoptotic stimuli

**Fig. 3:**
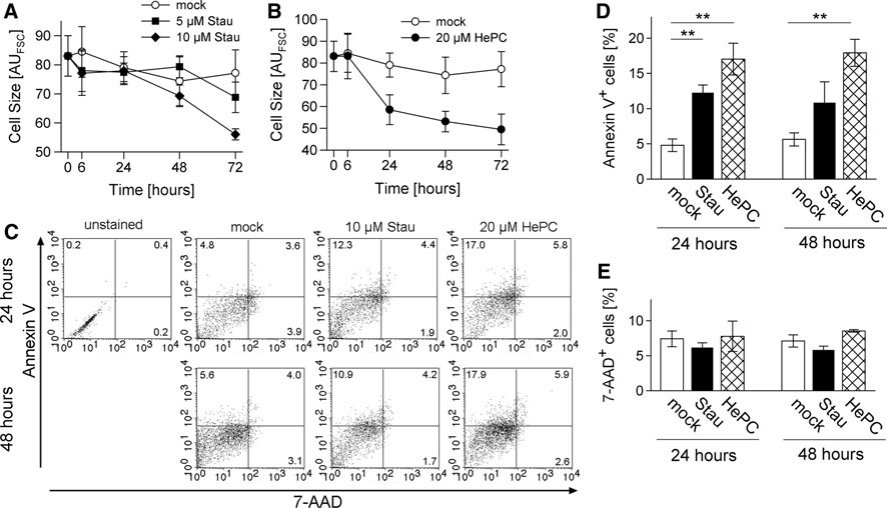
Features of apoptotic-like cell death in *T. gondii*. Parasites were treated with staurosporine (Stau) or miltefosine (HePC), or were mock-treated for the indicated periods of time. **a, b** Parasites were FACS-analysed and the mean forward scatter value was recorded as a relative measure of the cell size. Data represents mean ± SEM from at least three independent experiments. **c–e** Alternatively, parasites were labelled with Annexin V and 7-AAD to detect those which expose PS on their cell surface or those with a disrupted plasma membrane, respectively. They were then FACS-analysed; an unstained control was run in parallel. Two-dimensional dot plots from a representative experiment are depicted, percentages of single or double positive parasites are indicated (**c**). Mean percentages ± SEM of Annexin V^+^, 7-AAD^−^ (**d**) or 7-AAD^+^ parasites (**e**) were calculated from four independent experiments, significant differences between experimental groups are indicated (*p* < 0.01)

Besides DNA fragmentation, a reduction in cell volume (pyknosis) is a typical marker for apoptosis [18] and has also been extensively applied for detecting apoptotic protozoa [1]. Using flow cytometry, we therefore determined the cell size of *T. gondii* following treatment with pro-apoptotic stimuli. The results showed a time- and concentration-dependent decrease of the mean forward scatter (FSC) value following treatment of parasites with staurosporine (Fig. 3a). Miltefosine led to a decrease of the parasite cell size which was even more pronounced and occurred more rapidly than after staurosporine treatment (Fig. 3b). This time course was thus consistent with the stronger and accelerated induction of DNA strand breaks after treatment of *T. gondii* with miltefosine than after treatment with staurosporine (Fig. 1c, d).

We also determined the externalization of phosphatidylserine (PS) from the inner to the outer leaflet of the plasma membrane following treatment of *T. gondii* with pro-apoptotic stimuli. To this end, parasites were labelled with Annexin V and 7-AAD in order to distinguish between apoptotic and necrotic cells. The results showed that ~5 % of untreated parasites displayed PS on their plasma membrane suggesting some basal level of parasites undergoing an apoptosis-like cell death. Importantly, the percentages of Annexin V^+^, 7-AAD^-^ parasites clearly increased following treatment with staurosporine or miltefosine for 24 h as compared to mock-treated controls (Fig. 3c, d; *p* < 0.01, Student’s *t* test). This indicated the appearance of early apoptotic cells displaying PS on the cell surface but with an intact cell membrane. Between 24 and 48 h of treatment, the percentages of Annexin V^+^, 7-AAD^−^ parasites did not further increase but were again clearly higher as compared to mock-treated controls. During the time of observation, we observed only a minor increase in the percentages of 7-AAD^+^ cells after miltefosine treatment thus excluding a prominent induction of necrosis in *T. gondii* treated with staurosporine or miltefosine (Fig. 3c, e). Together, the data strongly suggested that following treatment with pro-apoptotic stimuli, *T. gondii* can undergo a form of programmed cell death that shows characteristic features of apoptosis as observed in metazoans.

### Identification of putative programmed cell death regulators in the *T. gondii* genome database

Molecules which might be involved in the regulation of an apoptosis-like cell death in *T. gondii* are yet unknown. In order to identify putative cell death regulators and executors, we performed a genome-wide in silico search of the *Toxoplasma* resource database (ToxoDB; Version 6.3) [26] for putative cell death-related genes. By employing a text-centered search approach, we identified 17 different *T. gondii* (strain ME49) genes with evidence of PCD-associated functions based on electronic annotation (Table 2). Genes retrieved in this manner encoded for proteins with sequence similarities to known or supposed regulators or executors of an apoptosis-like cell death in other unicellular eukaryotes including a putative endonuclease (TgME49_008710) [27, 28], several proteases including a putative metacaspase (TgME49_0078970, TgME49_093820, TgME49_049770, TgME49_006490) [29, 30], a defender against death (DAD)-like protein (TgME49_105870) [31] and two programmed cell death (PDCD) proteins (TgME49_094420, TgME49_105490) [5]. The autophagy regulator beclin 1 (the mammalian homologue of yeast Atg6) was also identified in *T. gondii* during this search (TgME49_021360) thus confirming the presence of an autophagy machinery [32] and autophagic cell death in *T. gondii* [33, 34].

**Table 2:** Putative cell death-related genes retrieved from the *Toxoplasma gondii* genomic database (http://toxodb.org/toxo/)

Gene ID	Name	Annotation
TgME49_008710	DNA/RNA non-specific endonuclease domain-containing protein	Nucleic acid binding/endonuclease activity/hydrolase activity/metal ion binding
TgME49_007620	Pyridine nucleotide-disulphide oxidoreductase domain-containing protein	Oxidation reduction/electron carrier activity/FAD binding/electron transport
TgME49_078970	ICE-like protease (caspase) p20 domain-containing protein	Proteolysis/cysteine-type endopeptidase activity/caspase activity
TgME49_053000	ELMO/CED12 family domain-containing protein	Phagocytosis
TgME49_094420	Programmed cell death protein, putative	No further information
TgME49_093820	Calpain family cysteine protease domain-containing protein	Proteolysis/calcium-dependent cysteine-type endopeptidase activity
TgME49_093830	Calpain, putative	Metabolic process/methyltransferase activity
TgME49_021360	Beclin-1, putative	Autophagy
TgME49_049770	Hypothetical protein, conserved	Homologue of TgVEG ‘Fas apoptotic inhibitory molecule, putative’
TgME49_109560	Hypothetical protein, conserved	Homologue of TgVEG ‘Fas apoptotic inhibitory molecule, putative’
TgME49_003030	Hypothetical protein	Homologue of TgVEG ‘Fas apoptotic inhibitory molecule, putative’
TgME49_105490	Programmed cell death protein, putative	No further information
TgME49_105870	Dolichyl-diphosphooligosaccharide-protein glycotransferase, putative	Homologue of TgGT1, TgVEG ‘defender against cell death, putative’
TgME49_035560	Hypothetical protein	Activation of caspase activity/protein binding/induction of apoptosis
TgME49_107780	Hypothetical protein	Proteolysis/calcium-dependent cysteine-type endopeptidase activity
TgME49_035880	Apoptosis-regulating basic protein, putative	No information
TgME49_006490	Metacaspase-1 precursor, putative	Proteolysis/cysteine-type endopeptidase activity/caspase activity

**Fig. 4:**
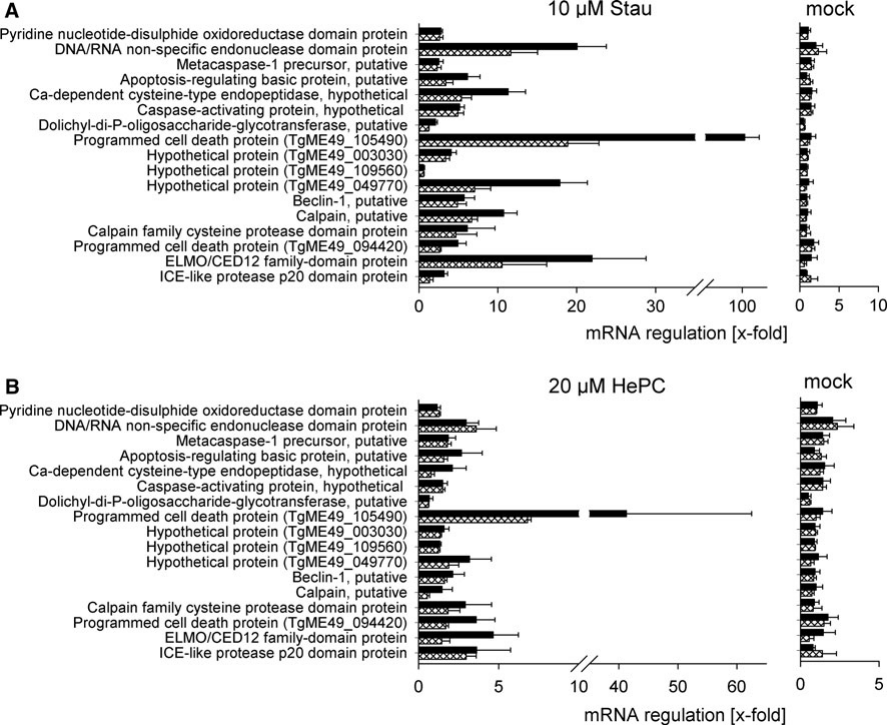
Transcript levels of putative PCD-related proteins after treatment of *T. gondii* with pro-apoptotic stimuli. Parasites were treated with staurosporine (Stau) or miltefosine (HePC), or were mock-treated for 24 h (*cross-hatched bars*) and 48 h (*black bars*). After RNA isolation and reverse transcription, cDNA was amplified by quantitative real-time PCR using primers specific for putative cell-death related genes and actin of *T. gondii*. Results are expressed as the mean ratio (*x*-fold) ± SEM of the mRNA after treatment as compared to freshly isolated, untreated parasites normalized to actin; data is from three independent experiments

To further explore a possible role of the proteins in apoptosis-like cell death in *T. gondii*, we compared the corresponding transcript levels following treatment of extracellular parasites with staurosporine and miltefosine by quantitative real-time RT-PCR. Transcripts of all 17 genes were detected in *T. gondii* and their expression did not change in mock-treated parasites after 24 or 48 h as compared to freshly isolated parasites (Fig. 4). In contrast, following treatment with staurosporine, the majority of mRNAs considerably increased in a time-dependent manner. Transcripts which were most strongly up-regulated encoded for a putative PDCD2 protein (TgME49_105490; ~100-fold up-regulated at 48 h), a putative ELMO and a DNA/RNA endonuclease (both more than 20-fold), a putative Fas apoptotic inhibitory molecule (~18-fold) and two calpain-like proteins (both more than 10-fold) (Fig. 4a). Transcripts of the putative PDCD2 protein TgME49_105490 also strongly increased after miltefosine treatment (more than 40-fold at 48 h), whereas others were only regulated to a minor extent or not at all (Fig. 4b). Together, the data suggest that *T. gondii* possesses ancient PCD machinery, the expression of which is differentially regulated during treatment with pro-apoptotic stimuli.

### Regulation of apoptosis-like cell death in *Toxoplasma*

**Fig. 5:**
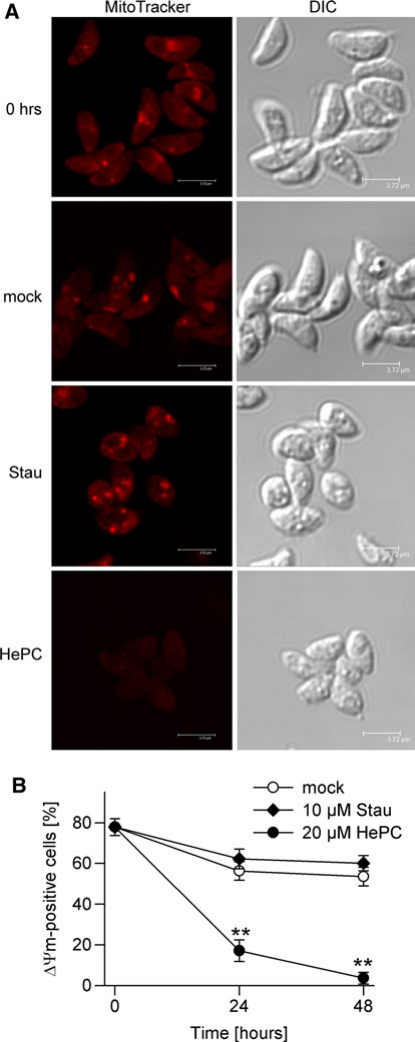
Loss of mitochondrial membrane potential (Δ*Ψ*_m_) during pro-apoptotic treatment of *T. gondii* with miltefosine. Parasites were treated with 10 μM staurosprine (Stau) or 20 μM miltefosine (HePC), or were mock-treated for 24 and 48 h. Freshly isolated parasites and treated parasites were incubated with MitoTracker probe Orange CM-H_2_TMRos. After fixation, cells were analysed by confocal laser scanning microscopy. **a** Representative images from three independent experiments are depicted. **b** The percentages of cells with an intact Δ*Ψ*_m_ were determined by counting at least 500 parasites per sample; data represents mean ± SEM from three independent experiments. Significant differences are indicated (**p < 0.01)

There is increasing evidence that mitochondria are not only useful marker organelles for detection of apoptosis in protozoa [1] but also play pivotal roles in the regulation of cell death pathways of protists [25, 27–29, 35, 36]. Therefore, we determined changes of the mitochondrial membrane potential (Δ*Ψ*_m_) after treatment of *T. gondii* tachyzoites with staurosporine and miltefosine. Mitochondria from freshly isolated parasites readily accumulated the MitoTracker probe thus indicating an intact Δ*Ψ*_m_ (Fig. 5a). Following treatment with 10 μM staurosporine for 48 h, the majority of parasites retained the Δ*Ψ*_m_ as also observed in mock-treated parasites. However, treatment with 20 μM miltefosine led to a loss of Δ*Ψ*_m_ in the majority of parasites (Fig. 5a). Time course analysis and enumeration of Δ*Ψ*_m_-positive cells revealed a significant decrease of parasites with functional mitochondria within 24 h of miltefosine treatment (*p* < 0.01; Student’s *t* test) with a further decrease thereafter (Fig. 5b). In contrast, staurosporine and mock treatments decreased the Δ*Ψ*_m_ by only 20 % and to a similar extent (*p* > 0.05). Thus, different cell death pathways appear to exist in *T. gondii*, one of which involves loss of Δ*Ψ*_m_.

**Fig. 6:**
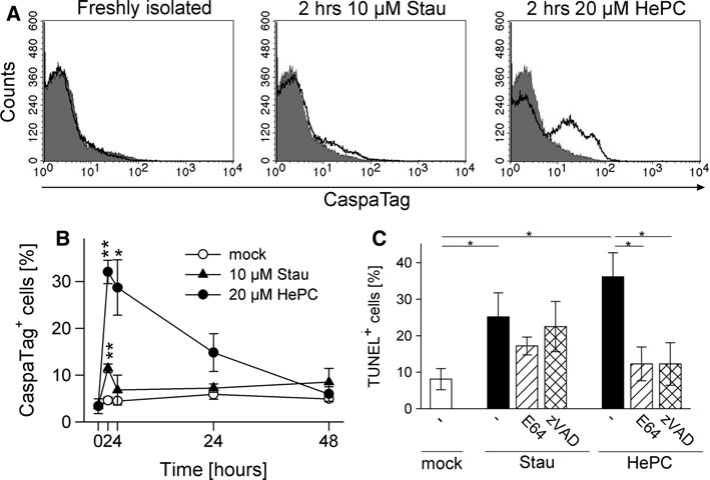
Activation of caspase-like proteases during pro-apoptotic treatment of *T. gondii*. Parasites were treated with 10 μM staurosporine (Stau) or 20 μM miltefosine (HePC), or were mock-treated for up to 48 h, or were freshly isolated. **a, b** Parasites were stained with a fluorescently labelled pan-caspase inhibitor (CaspaTag) and analysed by flow cytometry. **a** Representative FACS histograms of freshly isolated parasites or those treated for 2 h with pro-apoptotic stimuli (*open histograms*) as compared to mock-treated parasites (*filled histograms*). **b** Parasites were analysed at different time points post treatment and the percentage of CaspaTag-positive cells was determined. Data represents mean ± SEM from three independent experiments; significant differences between groups treated with pro-apoptotic stimuli or mock-treated are indicated (**p < 0.01; *p < 0.05). **c** Parasites were treated with protease inhibitors E64 or Z-VAD-FMK before being induced to undergo cell death with staurosporine or miltefosine. After 48 h, DNA strand breaks were visualized by TUNEL staining and positive cells were counted. Bars represent mean ± SEM from three independent experiments and significant differences are marked by asterisks (*p < 0.05)

Although protozoa do not express homologues of *bona fide* caspases [5], metacaspases or caspase-like activities have been recognized during PCD in several unicellular eukaryotes [21, 29, 30, 37, 38]. Furthermore, a putative metacaspase and several other proteases with possible roles in PCD have been identified in the *Toxoplasma* genome database (see above). We therefore tested the possibility that caspase-like proteases might be activated during cell death in *T. gondii*. In situ labelling of parasites with a caspase inhibitor and FACS analysis revealed a strong increase of binding within 2 h of miltefosine treatment (*p* < 0.001 as compared to mock-treated parasites; Student’s *t* test) (Fig. 6a, b). Such activity remained high at 4 h after miltefosine treatment before declining to control levels until 48 h post treatment. Staurosporine also induced a significant (*p* < 0.01) increase in caspase labelling within 2 h of treatment as compared to mock-treated controls (Fig 6a, b); however, inhibitor binding was much lower when compared to that observed in response to miltefosine and reached control levels already at 4 h of treatment. Mock-treated control parasites did not show any significant increase in caspase labelling (Fig. 6a, b).

In order to corroborate an involvement of caspase-like proteases in the miltefosine-triggered and staurosporine-triggered apoptosis-like pathways in *T. gondii*, parasites were pre-treated with cysteine protease inhibitor E64 or pan-caspase inhibitor Z-VAD-FMK before induction of cell death with staurosporine or miltefosine. As expected, treatment with both pro-apoptotic stimuli in the absence of protease inhibitors significantly increased the number of TUNEL-positive parasites (*p* < 0.05); however, in the presence of E64 or Z-VAD-FMK, occurrence of miltefosine-triggered DNA strand breaks was completely abolished (*p* < 0.05) (Fig. 6c). In contrast, both inhibitors did not significantly inhibit staurosporine-triggered DNA strand breaks although we observed a trend of E64 to partially reduce staurosporine-induced DNA cleavage. Together, this data suggests a distinct apoptosis-like cell death pathway in *T. gondii* which involves mitochondrial loss-of-function and caspase-like proteases and which is specifically activated by miltefosine but not staurosporine.

### Apoptosis-like cell death following treatment of *T. gondii* with parasiticidal drugs

**Fig. 7:**
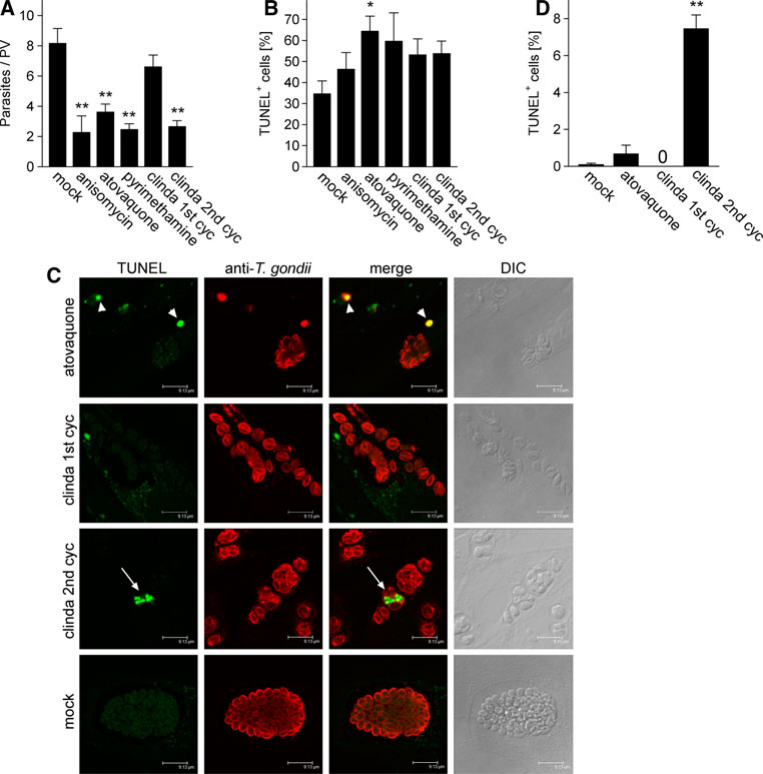
Toxoplasmacidal drugs can induce an apoptosis-like cell death in extracellular and intracellular *T. gondii*. Parasite-infected human fibroblasts were treated with 500 nM anisomycin, 100 nM atovaquone, 1 μM pyrimethamine or 500 nM clindamycin or were mock-treated. Clindamycin was applied during two consecutive cycles of parasite replication. After 48 h of treatment, parasites were immunofluorescently labelled (*red fluorescence*) and DNA strand breaks were visualized by in situ TUNEL labelling (*green fluorescence*). **a** Parasite replication was assessed by counting the number of parasites per 100 parasitophorous vacuoles (PV) and calculating the average parasite number per PV. *Bars* represent mean ± SEM from three independent experiments. **b, d** Extracellular (**b**) and intracellular (**d**) parasites that were positive following TUNEL labelling were counted; *bars* represent mean percentages ± SEM from three independent experiments. **c** Representative micrographs of cells treated with the indicated drugs for 48 h were obtained by confocal laser scanning microscopy. *Arrowheads* indicate TUNEL-positive extracellular parasites following atovaquone treatment and arrows indicate TUNEL-positive intracellular *T. gondii* during the second cycle of clindamycin treatment. Significant differences are marked by one (*p* < 0.05) or two *asterisks* (*p* < 0.01)

In order to test the hypothesis that apoptosis-like cell death might also play a role during chemotherapy of toxoplasmosis, *T. gondii*-infected human fibroblasts were treated with common anti-parasitic drugs and then assayed for occurrence of DNA strand breaks using TUNEL staining. Concomitant immunofluorescence labelling of parasites confirmed the significant reduction of parasite replication following treatments of *T. gondii* with 500 nM anisomycin, 100 nM atovaquone or 1 μM pyrimethamine as compared to the control treatment (*p* < 0.01) (Fig. 7a). Due to its delayed phenotype [17], 500 nM clindamycin effectively inhibited parasite replication only during the second (*p* < 0.01) but not the first replication cycle (Fig. 7a). The number of TUNEL-positive extracellular parasites increased after treatment with all chemotherapeutics for 48 h as compared to the mock treatment with the strongest increase being observed with atovaquone (*p* < 0.05) (Fig. 7b, c; marked by arrowheads in Fig. 7c). Remarkably, we also recognized a significant number of intracellular parasites which were strongly TUNEL-positive during the second cycle of clindamycin treatment (*p* < 0.001) (Fig. 7c, d; marked by arrows in Fig. 7c). Parasitophorous vacuoles (PVs) containing such TUNEL-positive parasites were found adjacent to PVs with TUNEL-negative parasites within the same host cell indicating a parasite-autonomous execution of the cell death program (Fig. 7c). In addition, they have lost their crescent shape and regularly contained multiple TUNEL-positive structures thereby resembling fragmented nuclei of mammalian cells undergoing apoptosis. TUNEL-positive intracellular parasites were not or only very rarely found during the first cycle of clindamycin treatment and during atovaquone treatment, respectively (Fig. 7d). Our data thus indicate that an apoptosis-like cell death can be triggered in extracellular and intracellular parasites undergoing drug treatment.

## Discussion

The mechanisms of how protozoa including several major pathogens of humans die are only recently being explored. This is astounding since they promise insights into the evolution of death pathways and may open up novel avenues to combat infectious diseases of high impact on human and animal health. Here we present several lines of evidence that strongly suggest the existence of a form of regulated cell death in the apicomplexan *T. gondii* that resembles apoptosis of higher eukaryotes. First, common markers of apoptosis including DNA strand breaks as detected by TUNEL assay, externalization of phosphatidylserine, reduction in cell volume and rounding-up of the cell were detected in the parasite. Second, *Toxoplasma* death could be triggered by staurosporine, miltefosine, hydrogen peroxide or camptothecin which also induce apoptosis in metazoan and other protozoa. Third, using in silico analysis of the *Toxoplasma* genome resource, we identified 17 genes with annotated functions in programmed cell death and which were differentially expressed following pro-apoptotic treatment of the parasite. Finally, *Toxoplasma* could be completely rescued from miltefosine-induced cell death by preventing the activation of cysteine proteinases. We therefore propose a form of cell death being present in *T. gondii* which—according to a recent classification of cell death by the Nomenclature Committee on Cell Death (NCCD)—resembles apoptosis of metazoans [18] and which we propose to refer to as apoptosis-like cell death. Besides the detection of several common apoptosis markers in *T. gondii*, this term takes into consideration that molecular pathways which characterize apoptosis in higher eukaryotes [39] only partially overlap with those present in *T. gondii*. Our findings on an apoptosis-like cell death in *Toxoplasma* are consistent with previous reports on the occurrence of DNA fragmentation, hypoploid nuclei and condensation of chromatin after treatment of *T. gondii* with the nitric oxide donor sodium nitroprusside [40]. They are also in line with the presence of ancestral PCD pathways in several divergent branches of unicellular eukaryotes including the Apicomplexa and an early origin of PCD during evolution [2, 3, 5, 6].

A major question arising from our finding concerns the functional significance of apoptosis-like cell death in *T. gondii*. Toxoplasms in a distinct host are considered a clonal population since they originate from a single inoculum transferred by ingestion of undercooked meat from a chronically infected live stock or of oocysts shed by cats into the environment [7]. In addition, sexual reproduction only takes place in the intestinal epithelium of *Felidae* but not in humans or other vertebrates. Finally, *T. gondii* infection elicits a robust Th1-type immunity that allows persistence of parasites from the primary infection but only rarely those from a *Toxoplasma* super-infection. However, as a population of genetically identical organisms in a distinct host, the programmed cell death of a subpopulation of parasites may be evolutionary sustainable because it could help avoiding an overwhelming parasite load and host death before parasite transmission would have been possible [41]. This may be particularly important for *T. gondii* the transmission cycle of which critically depends on establishing a long-lasting chronic infection. A parasite population exposing phosphatidylserine on the outer leaflet of the plasma membrane has indeed been described in the peritoneal fluid of mice during acute infection [42]. Although these parasites appeared to be TUNEL-negative they are nevertheless reminiscent of apoptotic cells and could have contributed to regulate the parasite density during acute infection. Whether such suicide-mediated cell death also regulates parasite numbers during chronic infection awaits clarification. Furthermore, apoptosis-like cell death and exposure of phosphatidylserine by *T. gondii* has been reported to dampen the host immune response by inducing TGF-β secretion, i.e. a mechanism known as apoptotic mimicry [42, 43]. Future studies are, however, clearly needed to more precisely determine the phenotype of phosphatidylserine-exposing parasites from the peritoneal cavity. Furthermore, the molecular mechanisms leading to this phenotype are unknown.

Here, we present evidence that different apoptosis-like cell death pathways were induced in *T. gondii* in response to staurosporine and miltefosine treatments (Table 3). One of these pathways involved a rapid and strong activation of caspase-like proteases, a loss of the Δ*Ψ*_m_, and high expression levels of a putative member of the PDCD2 superfamily of pro-apoptotic regulators. Permeabilization of the outer mitochondrial membrane (MOMP) is crucial in integrating various upstream signals into the caspase 9-caspase 3/7 cascade in mammals but also into the release of caspase-independent pro-apoptotic factors including EndoG or AIF [44]. The Δ*Ψ*_m_ collapses shortly after MOMP in various mammalian systems although its contribution in promoting apoptosis is still controversial [45]. Dissipation of Δ*Ψ*_m_ has been linked to apoptosis-like cell death in *Leishmania* and *Trypanosoma* parasites [29, 46–48], and in the free-living slime mold *Dictyostelium discoideum* [35] while its involvement in *Plasmodium* apoptotic pathways is a matter of debate (reviewed in [1]). In *T. gondii*, Δ*Ψ*_m_ was rapidly lost following miltefosine treatment indicating mitochondrial dysfunction in dying parasites. However, whether this is an essential step in the regulation and execution of miltefosine-induced cell death in *T. gondii* awaits future clarification. In contrast, cysteine protease activity was clearly required during miltefosine-induced cell death in *T. gondii* since E64 and Z-VAD-FMK completely abolished drug-induced TUNEL positivity. A critical role of caspase-like proteases in this pathway is further supported by the strong increase of CaspaTag-positive parasites within two hours of miltefosine treatment. The CaspaTag assay used herein relies on binding of a fluorochrome-labelled inhibitor of pan-caspases, FAM-VAD-FMK, to activated caspases. However, VAD-FMK does not exclusively bind *bona fide* caspases but also other cysteine proteases including distinct cathepsins (i.e., clan CA cysteine proteases) and legumain (belonging to clan CD cysteine proteases, which also comprise caspases and metacaspases) [49]. This explains the efficient binding of VAD-FMK inhibitors in miltefosine-treated *T. gondii* despite absence of an authentic caspase in the parasite genome ([5] and our own bioinformatic analysis). Several clan CA cysteine proteases including cathepsin B, L and C proteases have been characterized in *T. gondii* and play crucial roles in maturation of excretory/secretory proteins, host cell invasion and replication [50–52]. A role in PCD has not yet been reported for any of these proteins. In our bioinformatic search, we identified several additional proteases which might fulfil pro-apoptotic functions in *T. gondii* including a metacaspase-1 (TgME49_006490), an ICE-like protease (TgME49_078970) and several calpain-type cysteine proteases (TgME49_093830, TgME49_093820, TgME49_107780). Distinct mammalian calpains regulate apoptosis (reviewed in [53]), and metacaspases of some protozoan parasites have been implicated in PCD [30, 37, 38]. It will thus be interesting to see whether one of the newly identified PCD-related proteases of *T. gondii* is responsible for the miltefosine-induced apoptosis-like cell death.

**Table 3:** Regulation of apoptosis-like cell death pathways in *T. gondii* triggered by pro-apoptotic stimuli

	Staurosporine	Miltefosine
Mitochondria	Intact Δ*Ψ*_m_	Loss of Δ*Ψ*_m_
Caspase-like activity	Small but significant increase at 2 h	Strong increase at 2 and 4 h
Protease inhibitors (E64, zVAD)	No significant impact	Prevention of cell death
Up-regulation of mRNAs (>10-fold)	PDCD2, ELMO, DNA/RNA endonuclease, Fas apoptotic inhibitor, calpain-like proteins	PDCD2
Downstream apoptotic markers	DNA strand breaks (TUNEL), PS exposure, no membrane permeabilization, pyknosis, rounding-up of cell	DNA strand breaks (TUNEL), PS exposure, no membrane permeabilization, pyknosis, rounding-up of cell

Another feature of miltefosine-induced *Toxoplasma* cell death was the strong up-regulation of a putative PDCD2 family member (Table 3). Increased PDCD2 expression can induce apoptosis in human cells, at least in part by activating a caspase cascade, whereas repression of PDCD2 is involved in the pathogenesis of certain human lymphomas [54, 55]. It is evolutionarily conserved and has been identified in all eukaryotes [5]. Here, we confirm that it is also present in the *Toxoplasma* genome and—more importantly—we show that it is strongly up-regulated in response to pro-apoptotic stimuli. Pro-apoptotic treatment of rat thymocytes increased the expression of Rp8, i.e. the rat orthologue of PDCD2 and this preceded DNA fragmentation [56]. This supports the hypothesis that the pro-apoptotic function of PDCD2 is transcriptionally regulated. Whether its pro-apoptotic effect is due to repression of gene transcription by interaction with N-CoR/mSin3A corepressor complexes [57, 58] and/or activation of a caspase cascade [55] remains to be clarified. In *T. gondii*, up-regulation of PDCD2 did not correlate with activation of caspase-like proteases, since staurosporine induced high levels of PDCD2 mRNA but only a minor (though significant) and transient activation of caspase-like proteases. It should be mentioned that PDCD2 in *T. gondii*, as in most other unicellular eukaryotes [5], contains the C-terminal PDCD2 domain (IPR007320) but not the N-terminal zinc finger, MYND-type domain that is present in PDCD2 of higher eukaryotes. In humans, the MYND domain of PDCD2 interacts with cell cycle regulator host cell factor 1 (HCF-1) and presumably also with N-Cor/mSin3A complexes [57, 58], but it is unknown whether these or other interactions are required for the pro-apoptotic function of PDCD2. Thus, despite the absence of MYND in PDCD2 from *T. gondii*, it can well be that it exerts pro-apoptotic functions. However, the exact role and molecular mechanisms of PDCD2 during cell death in *T. gondii* requires further investigation.

Interestingly, high PDCD2 mRNA levels were also observed after staurosporine treatment whereas—in contrast to the response to miltefosine—parasites retained an intact Δ*Ψ*_m_ and showed considerably less caspase-like activity (Table 3). It should be stressed that the intact Δ*Ψ*_m_ does not exclude an involvement of mitochondria in staurosporine-triggered apoptosis-like cell death in *T. gondii* since loss of Δ*Ψ*_m_ is also not necessarily crucial for MOMP in mammals [45] and for execution of apoptosis in invertebrates [44, 59]. It nevertheless indicates clear differences in the signalling pathways that are activated in *T. gondii* in response to different pro-apoptotic stimuli. This view is further sustained by a much more complex transcriptional response to staurosporine as compared to that observed in response to miltefosine. Thus, despite the fact that apoptotic pathways in unicellular eukaryotes are considered ancestral, they nevertheless appear to be highly complex.

Here, we also present the first report that treatment of *T. gondii* with drugs which are commonly used for chemotherapy of toxoplasmosis, can induce a form of parasite death that show signs of apoptosis. This finding is of major interest because it opens up the possibility to target endogenous cell death pathways of *T. gondii* in order to combat toxoplasmosis. DNA strand breaks were most strongly induced in extracellular parasites using the hydroxy-naphthoquinone atovaquone which targets the cytochrome *bc*_*1*_ complex thereby inhibiting the mitochondrial respiratory chain [60]. In contrast, atovaquone induced only very low levels of DNA fragmentation in intracellular parasites. Intriguingly, however, a significant number of intracellular parasites showed signs of apoptosis following treatment with the lincosamide clindamycin. Clindamycin inhibits protein synthesis of the apicoplast, a non-photosynthetic plastid of apicomplexan parasites [61], by binding to rRNA of the large subunit [62]. Consistent with the well-known delayed parasiticidal activity of clindamycin ([17]; Fig. 7a), DNA fragmentation in intracellular parasites only occurred during the second cycle of replication and this coincided with a significant reduction of the parasitic load. It should be mentioned that only a minor portion of intracellular *T. gondii* presented signs of apoptosis following clindamycin treatment. This is unlikely due to local differences in drug concentrations since vacuoles with TUNEL-positive parasites were adjacent to vacuoles with TUNEL-negative parasites within the same host cell. It may rather result from the fact that *T. gondii* is particularly vulnerable to clindamycin during the late stages of endodyogeny, i.e. the formation of two daughter cells from the mother cell [62]. This would indicate the induction of an apoptosis-like cell death by clindamycin during distinct stages of parasite replication. Furthermore, the additive effect of the drug during successive rounds of the parasites’ lytic cycle [63] suggests that the number of TUNEL-positive parasites may further increase during prolonged drug treatment and may have been not detected in our experiments until 48 h of the second cycle of treatment. In summary, an apoptosis-like cell death can be triggered in intracellular *T. gondii* by chemotherapeutic agents that specifically target the parasite as shown herein. Detailed knowledge of the apoptosis-like death pathway induced by clindamycin and possible other parasiticidal drugs might be exploited in the future to combat *Toxoplasma* and related parasites, e.g. the malaria parasite *Plasmodium* more effectively.
